# Using google street view for systematic observation of the built environment: analysis of spatio-temporal instability of imagery dates

**DOI:** 10.1186/1476-072X-12-53

**Published:** 2013-12-03

**Authors:** Jacqueline W Curtis, Andrew Curtis, Jennifer Mapes, Andrea B Szell, Adam Cinderich

**Affiliations:** 1 GIS Health & Hazards Lab, Department of Geography, Kent State University, Kent, OH 44242, USA; 2 Department of Geography, Kent State University, Kent, OH 44242, USA

**Keywords:** Google Street View (GSV), Built environment audit, Date, Imagery, Spatial video

## Abstract

**Background:**

Recently, Google Street View (GSV) has been examined as a tool for remotely conducting systematic observation of the built environment. Studies have found it offers benefits over in-person audits, including efficiency, safety, cost, and the potential to expand built environment research to larger areas and more places globally. However, one limitation has been the lack of documentation on the date of imagery collection. In 2011, Google began placing a date stamp on images which now enables investigation of this concern. This study questions the spatio-temporal stability in the GSV date stamp. Specifically, is the imagery collected contemporaneously? If not, how frequently and where is imagery from different time periods woven together to represent environmental conditions in a particular place. Furthermore, how much continuity exists in imagery for a particular time period? Answering these questions will provide guidance on the use of GSV as a tool for built environment audits.

**Methods:**

GSV was used to virtually “drive” five sites that are a part of the authors’ ongoing studies. Each street in the sites was “driven” one mouse-click at a time while observing the date stamp on each image. Every time the date stamp changed, this “disruption” was marked on the map. Every street segment in the site was coded by the date the imagery for that segment was collected. Spatial query and descriptive statistics were applied to understand the spatio-temporal patterns of imagery dates.

**Results:**

Spatio-temporal instability is present in the dates of GSV imagery. Of the 353 disruptions, 82.4% occur close to (<25 m) intersections. The remainder occurs inconsistently in other locations. The extent of continuity for a set of images collected with the same date stamp ranged from 3.13 m to 3373.06 m, though the majority of continuous segments were less than 400 m.

**Conclusion:**

GSV offers some benefits over traditional built environment audits. However, this investigation empirically identifies a previously undocumented limitation in its application for research. Imagery dates can change often and without warning. Caution should be used at intersections where these disruptions are most likely to occur, though caution should be used everywhere when using GSV as a data collection tool.

## Background

### Introduction

Systematic observation of the built environment is an important tool for collecting fine scale spatial data on the conditions in which people live and on the role of place in shaping health [1]. Though this approach is not new, it has recently received renewed attention due to advances in geospatial technologies that enable researchers to conduct these audits remotely. This development has been proposed as a way to save time and money, to improve safety of research personnel, and to expand the scale of these audits to cover larger areas and more study sites around the world [2-8]. Ideally, such improvements will lead to greater understanding of the relationship between the built environment and health, with the ultimate aim of improving health outcomes.

Despite the potential of remote audits, the geospatial technologies that make them possible are relatively new and relatively little is known about them as research tools. For example, the most commonly used source for built environment audits is Google Street View (GSV). It has only been in existence since 2007 and its purpose is not research and certainly not built environment audits. To date, the focus of studies that use this approach has been on the reliability of the tool when comparing its use with in-person audits and among many raters using one instrument remotely (inter-rater reliability). Based on these metrics alone, remote audits have been demonstrated to be reliable with some exceptions such as with capturing visually small and temporally dynamic characteristics like litter or graffiti, and human activities. Overall, existing studies that use GSV and similar technologies for remote audits have touted their potential and noted only minor limitations. One of these limitations has been the lack of a date stamp on the imagery [2,3,5-10]. Until 2011, however, this limitation was only a supposition as no documentation of date of imagery collection was provided by Google. In 2011, GSV introduced a date stamp (month and year) on its imagery. Prior to this point, remote audits were being performed with uncertainty on exactly when the data were collected or had to request this information from the proprietor. Perhaps for many study sites, the date of imagery collection does not matter as most characteristics of the built environment do not change much from year to year. However, this is an uncomfortable assumption and one that reduces the rigor of built environment research. Furthermore, for the emerging studies that investigate the health impact of policies focused on changing the built environment, such as blight removal or neighborhood recovery after a natural disaster, a dynamic landscape is expected and quantifying its change is central to the research. Knowing the date of imagery is essential.

The 2011 addition of month and year date stamp to each GSV image permits a more comprehensive understanding of the appropriateness of this technology for research. With these data, it is no longer necessary to speculate about when the imagery were collected for study sites, nor merely note this uncertainty as a possible limitation. This paper presents findings from the first documentation and analysis of spatio-temporal patterns in the new month-year date stamp on GSV imagery. Results clearly indicate the need to perform analysis of these meta-data when GSV is used as a source of systematic observation of the built environment.

### The brief history of freely available online geospatial technologies for remote built environment audits

Systematic observation of the built environment yields data at fine spatial scales (e.g., individual point locations of litter, blighted structures). These data have received considerable attention due to their ability to explain the role of place in health [11-14] and safety [15-17], though the majority of research using this approach has been conducted under the auspices of “active living” and physical activity [18]. Despite accepted use of the approach, a recent review conducted by Schaefer-McDaniel and colleagues [19] find that it has been employed with such variability that this lack of rigor has led to inconsistent findings in understanding the relationship between the built environment and health.

Though systematic observation was traditionally conducted by walking a study area, advances in technology, particularly with video have been employed [20]. Further improvements in the size, cost, and ease of use of geospatial technologies such as GPS have led to its integration in these surveys as well. For example, GPS-enabled video allows researchers to drive study areas and collect geotagged video which can be “driven” in a Geographic Information System (GIS) to facilitate coding the built environment for further spatial analysis [21-24]. Recently, Oliver and colleagues [25] introduced another technological advance, SenseCam, which enables people to walk through study areas with an unobtrusive GPS-enabled video camera attached to a lanyard around their neck. However, such field-based data collection approaches are not without their limitations, such as financial and time investments, as well as safety of those collecting the data. Given these constraints, it makes sense that GSV and similar online technologies are investigated as a proxy for actually going into the field to conduct environmental audits. Indeed, this subject is receiving growing attention as evidenced by the increasing numbers of articles on the subject from two each year from 2010–2012 to five in the first half of 2013 alone.^a^

Badland and colleagues [2] were the first to publish on the potential use of GSV as a tool for conducting neighborhood audits. The authors used the NZ-SPACES tool to collect built environment data on a neighborhood in person, and then virtually using GSV, within a week later. Overall, this study finds good agreement between the physical and virtual audits and proclaims the potential of GSV for studies on the built environment as it relates to health. They identify one limitation with the approach, the time differential between date of imagery collection and date of field data collection. They note that the images were collected “between January and July 2008” (1011). This information was acquired through a personal communication with a Google public relations manager. Furthermore, another Google employee informed the authors that “It is anticipated that Google will update Street View images every 18 months” (1014).^b^

Subsequent studies in the past three years have produced similar findings regarding the benefits and limitations of using GSV for virtual built environment audits. Overall, they find the following:

a) There is good agreement between virtual and physical audits, especially for large, visible features [2,3,7-10,26].

b) Remote audits are less effective for small features, those that necessitate qualitative decisions (e.g., sidewalk conditions), social activities [7], and those that are temporally variable [3,4,6,8-10,26].

c) Remote audits reduce the financial and human resource cost of neighborhood surveys, which can limit their use [3,4,6-9].

In addition to these findings in common, several studies highlight individual issues that they discovered in the course of their audits. For example, Ben-Joseph and colleagues propose that on-site audits offer context for what is being viewed, rather than just dropping directly in to a study area [9]. Kelly and colleagues note that imagery is not available for all streets [10]. Taylor and colleagues observe that view and quality of the imagery in remote audits can also pose limitations [4]. Related to imagery dates, several studies did mention that a temporal lag between imagery date and in-person audits may be problematic [3,4,26]. Indeed, Clarke and her colleagues observe that “the dates of the images in Google Street View are not always readily apparent.” (1227).^c^ Clarke and Gallagher [27] then raise the possibility that “temporal mismatch may have contributed to some misspecification in our models and constrained the ability to detect stronger effects in the urban accessibility measure”.

Overall, these studies agree that GSV is reliable as a remote audit tool, but that the uncertainty of the date of imagery is a potential limitation. With the newly implemented date stamps on GSV imagery, this limitation can be examined empirically to understand the extent to which researchers should be concerned and to then propose how to address these limits in methodology. It is with this background and justification that this study questions the spatio-temporal stability in the GSV date stamp. Specifically, for five study sites, is the imagery collected contemporaneously? If not, how frequently and where is imagery from different time periods woven together to represent environmental conditions in a particular place. Furthermore, how much continuity exists in imagery for a particular time period? Answers to these questions will provide guidance to researchers on the appropriate use of Google Street View as a remote tool for systematic observation of the built environment.

## Results and discussion

### *Disruption*

**Figure 1:**
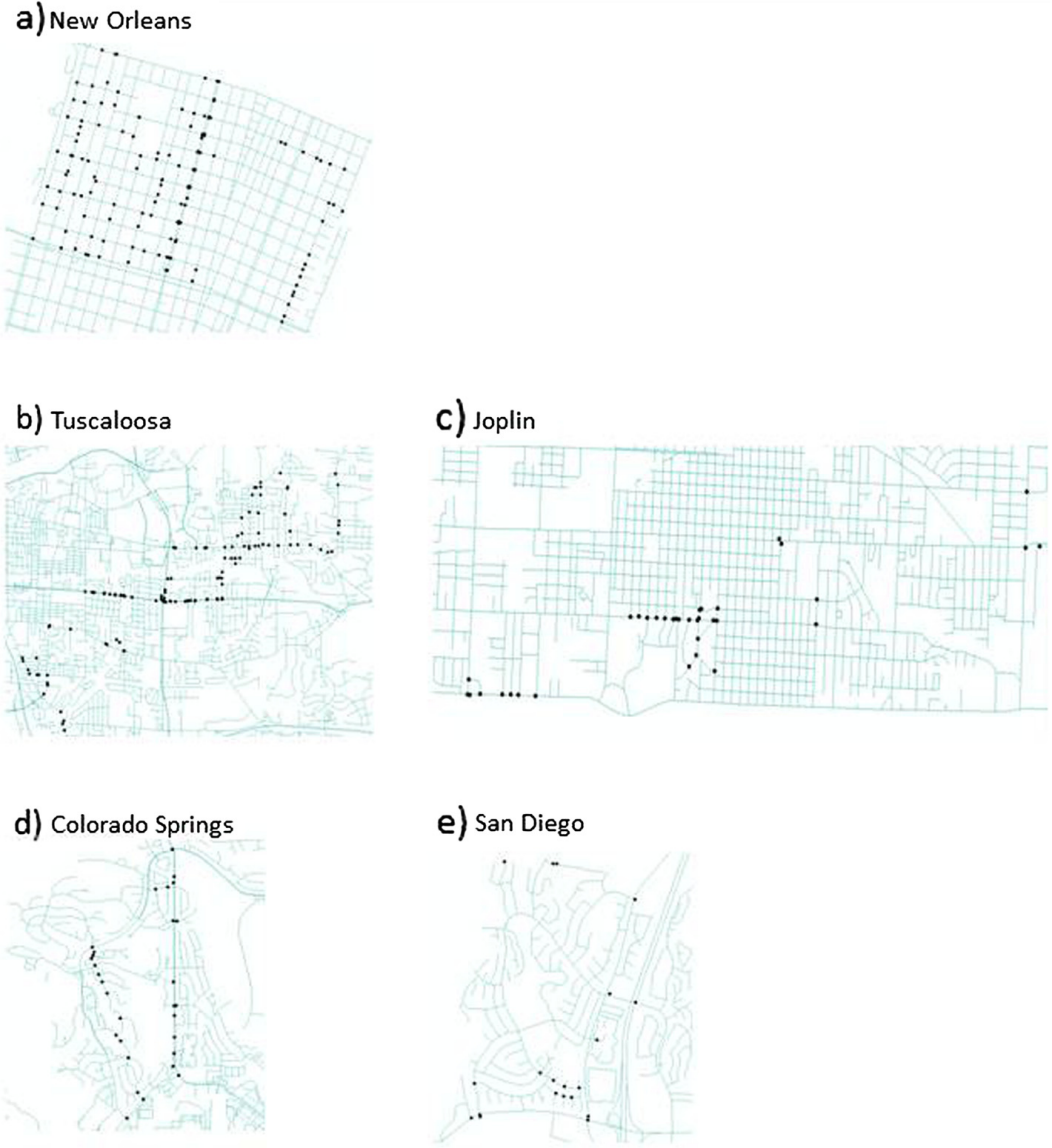
**Locations of disruptions in the five study sites.** Each black point is a location of disruption in the study sites: **a)** New Orleans, **b)** Tuscaloosa, **c)** Joplin, **d)** Colorado Springs, and **e)** San Diego.

The date of GSV imagery changed 353 times across the five study sites (Figure 1). From observations recorded while identifying these locations, it was noted that many of the date changes occurred within or near street intersections. Therefore, a spatial query was employed to measure the distance from each point to its nearest intersection. Results of the spatial query reveal that of these points, 291 (82.44%) occur within 25 m of an intersection (Table 1). This spatial pattern of disruptions within 25 m of intersections is present in all of the study areas individually as well, ranging from a low of 70.0% in San Diego to a high of 94.3% in Joplin (Table 1). However, of these disruptions, the majority occur at even smaller distances from the closest intersection, most within 10 m (71.7%) (Table 2).

**Table 1:** Location of imagery date disruptions from the nearest intersection

**Distance from intersection**	**Total number of disruptions (%)**	**New Orleans (%)**	**Tuscaloosa (%)**	**Joplin (%)**	**Colorado Springs (%)**	**San Diego (%)**
0-25 m	291	118	104	33	22	14
	(82.4%)	(87.4%)	(80.0%)	(94.3%)	(73.3%)	(70.0%)
25.01-50 m	44	15	20	2	4	3
	(12.5%)	(11.1%)	(15.4%)	(5.7%)	(13.3%)	(15.0%)
50.01-75 m	5	1	3	0	1	0
	(1.4%)	(0.7%)	(2.3%)	(0%)	(3.3%)	(0.0%)
75.01-100 m	6	1	0	0	1	1
	(1.7%)	(0.7%)	(0%)	(0%)	(3.3%)	(5.0%)
>100 m	7	0	3	0	2	2
	(2%)	(0%)	(2.3%)	(0%)	(6.7%)	(10.0%)
Total disruptions by study site	353	135	130	35	30	20

(Total number of disruptions, n = 353).

**Table 2:** Location of imagery date disruptions from the nearest intersection for the subcategory of 0-25 m

**Distance from intersection**	**Number of disruptions (%)**
<0.01 m	65
	(22.3%)
0.01-5 m	63
	(21.6%)
5.01-10 m	81
	(27.8%)
10.01-15 m	39
	(13.4%)
15.01-20 m	22
	(7.6%)
20.01-25 m	21
	(7.2%)

(Total number of disruptions in this subcategory, n = 291).

### *Continuity*

Given that disruptions in imagery date are present in all five study sites, and particularly near intersections, the next question was the extent of continuity in imagery dates between these disruptions. Specifically, how much continuity exists in imagery for a particular time period? Table 3 demonstrates that continuity of imagery date was present for road segments 3.13 m long to 3373.06 m long for the study as a whole, and also shows the variability that exists among the five sites. These relatively small areas are represented by imagery from different dates covering different lengths of road segments woven together to present a seamless picture of place at a particular time. However, in reality, imagery from multiple time periods is present, even though this variation is not readily apparent to the viewer. This situation becomes evident when investigating the length of each road segment by the date in which imagery for it is presented.

**Table 3:** Imagery date disruptions and extent of continuity in each study area

**Study area**	**Study area size (km**^**2**^**)**	**Roads in study area (m)**	**Number of intersections**	**Number of disruptions**	**Extent of continuity (m)**
Lower Ninth Ward, New Orleans, Louisiana	2.77	51951.53	1189	135	3.71-1928.13
(Hurricane Katrina, August 29, 2005)					
Tuscaloosa, Alabama	15.03	50857.19	1468	130	3.13-1494.97
(EF5 tornado, April 27, 2011)					
Joplin, Missouri	11.20	99121.25	1572	35	4.85-3373.06
(EF5 tornado, May 22, 2011)					
Mountain Shadows, Colorado Springs, Colorado	5.30	48543.74	653	30	7.55-2720.24
(Waldo Canyon Wildfire, June 23, 2012)					
Rancho Bernardo, San Diego County, California	4.85	31117.95	403	20	17.37-2297.56
(Witch Wildfire, October 21, 2007)					

**Figure 2:**
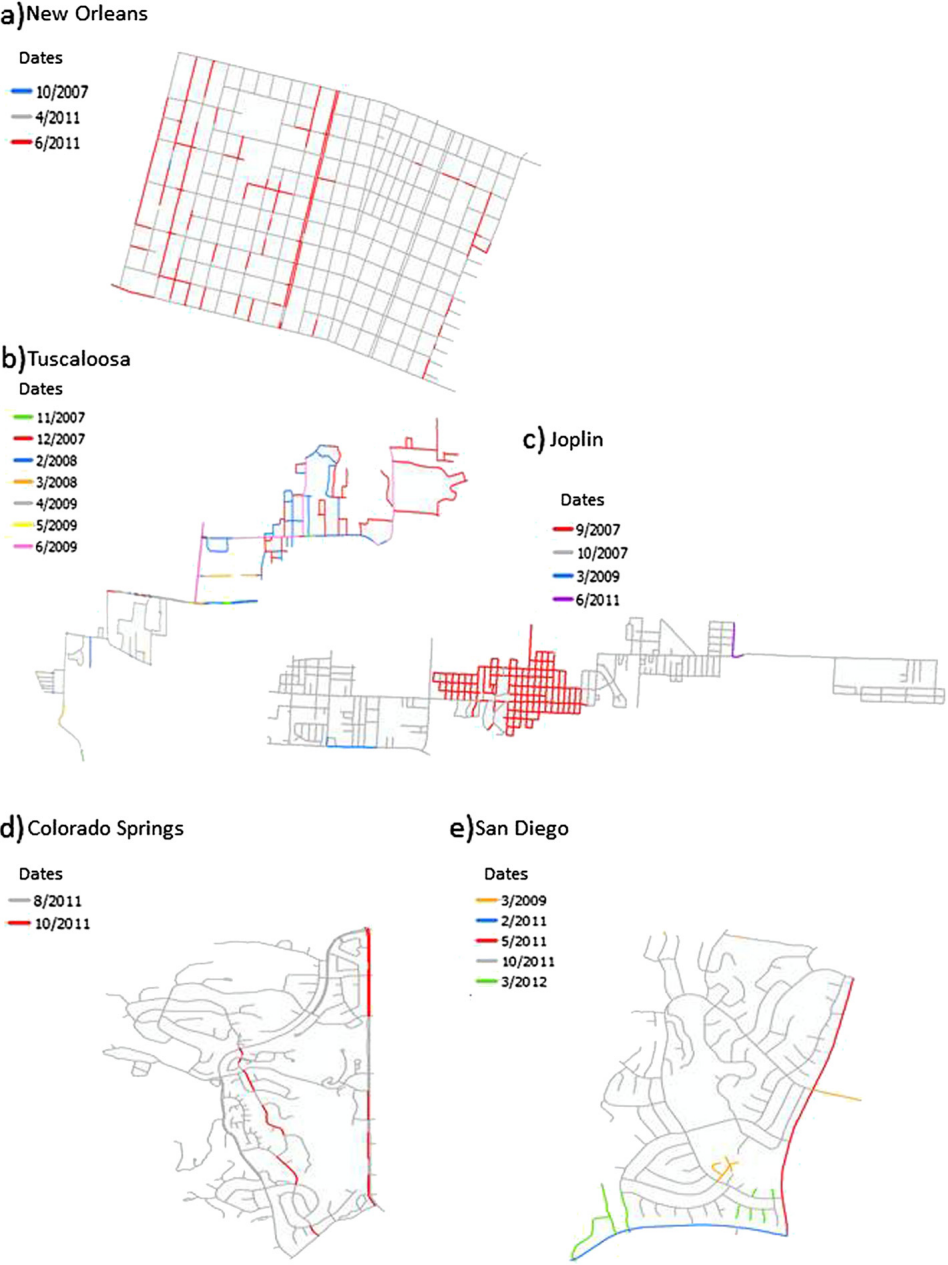
**Spatial patterns of continuity for the five study sites.** For each site, the dates of GSV imagery are listed chronologically. The dates are color-coded based on their percentage of all dates represented in the study site, with gray being the color of the majority date stamp. Each of the study sites is represented: **a)** New Orleans, **b)** Tuscaloosa, **c)** Joplin, **d)** Colorado Springs, and **e)** San Diego.

For all study areas, except San Diego and Colorado Springs, imagery for one date can be presented for less than 5 m and then change to imagery from another date. The minimum in San Diego is approximately 17 m and the minimum in Colorado Springs is approximately 7 m. However, imagery from a single date stamp can also be presented for as long as 1494.97 (Tuscaloosa) or 3373.06 m (Joplin). As demonstrated in Figure 2, the spatial pattern of imagery dates is variable across the study sites. In all maps in this figure, gray represents roads collected on the majority date stamp according to GSV. For example, in the New Orleans study area, the majority of the imagery was collected in 4/2011, so all road segments with imagery collected for this date are colored gray. The second most common date of imagery is 6/2011, shown in red. One small segment (47.66 m) was collected in 10/2007 and it is highlighted in blue. However, the Tuscaloosa study area stands in stark contrast with imagery date disruptions occurring frequently and, in many cases, lasting for only a few meters.

### Implications

**Figure 3:**
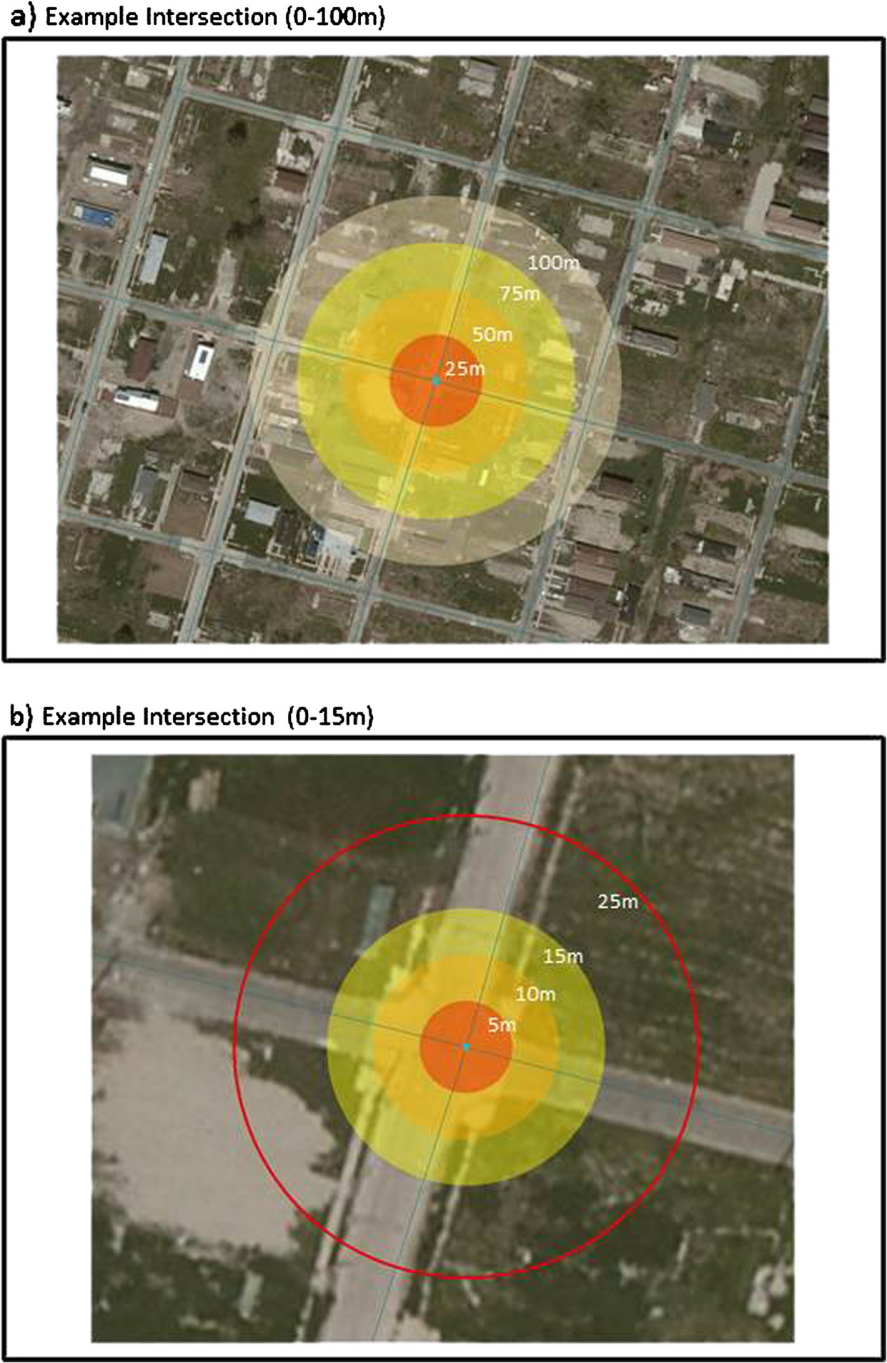
**The context of disruption. a)** For a sample intersection, the centroid of the intersection was buffered at 25 m, 50 m, 75 m, and 100 m. **b)** Then, for the same intersection, the centroid was buffered at 5 m, 10 m, 15 m, and 15 m.

Identifying the location and duration of imagery date disruptions in GSV for these five study sites clearly indicates that caution must be used at intersections when employing this geospatial technology to conduct built environment audits. Imagery date disruptions most often occur within 25 m of intersections (82.4%) and of these, the majority is found within 10 m from the intersection (71.7%).^d^ Why do these distances matter? Figure 3 demonstrates the context of these distances for an example intersection. The 10 m buffer around the center of the intersection shows that this distance approximately covers an intersection from entrance through exit. This is a small distance which could easily be scrolled through with a mouse, not realizing that the date of imagery has changed from what it was prior to entering the intersection to what it becomes when exiting the intersection.

**Figure 4:**
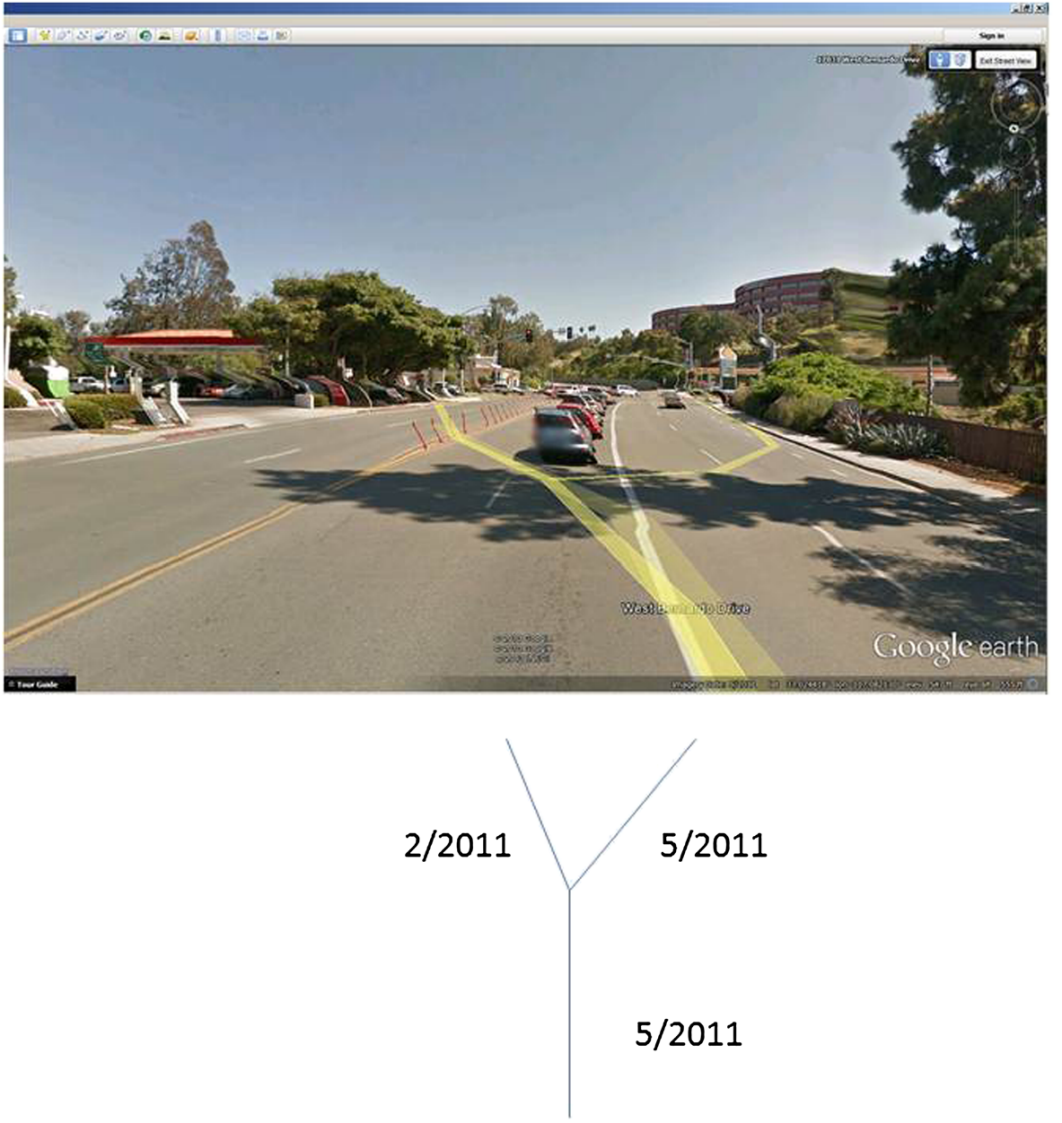
**The context of continuity.** The imagery for the main segment of this road was collected during May 2011. The yellow GSV path then splits just prior to the intersection. The imagery for the right path was collected during May 2011, but imagery for the left path was collected three months earlier in February 2011.

However, there is also evidence of disruptions within road segments at other locations, such as in the middle of a block or in a cul-de-sac. Therefore, while intersections present the most problematic geographic feature, caution really must be used at all times with GSV as a data collection tool. Furthermore, continuity in imagery date is variable, as demonstrated in Figure 2. It is especially concerning that approximately 37% of continuous road segments measure 100 m or less. This distance is roughly the length of a city block in the New Orleans study site. When scrolling through the street imagery using a mouse, it is entirely possible to not notice the small, quick changes in the dates of imagery collection. Furthermore, these disruptions can occur where otherwise longer extent of continuity exists. This situation is particularly true with small splits in the GSV path within one road segment, as demonstrated in the San Diego study site (Figure 4).

The results of this study present a caution to the research community about relying on the imagery provided through GSV and similar corporate enterprises. These are secondary data with all of the concerns and caveats that apply to data collected for any purpose other than that specified by the research at hand. However, given the relative novelty of these data, a better understanding of its benefits and limitations is especially needed. Results of this study suggest that it would be wise to take note of the date stamp when using GSV to perform systematic observation of the built environment. Granted, though all environments are dynamic, some are more dynamic than others. It is therefore incumbent on researchers to assess the importance of the date of imagery collection on a case by case basis and to understand the patterns of continuity and disruption in their study areas. Second, the fact that the patterns identified in this study are temporally ephemeral as Google replaces GSV data also argues for the need for researchers to archive the GSV data they use. No one should operate under the assumption that these data will be held in perpetuity and continue to be made publicly available.

### Limitations

Despite the contributions of this paper, namely the identification of potential sources of error due to imagery date disruption, it is not without its limitations. First, only five study sites are examined. They are small and vary by type (suburban and urban) and geographic location in the United States. Perhaps these areas represent anomalies in the temporal stability of GSV data. Certainly, only a convenience sampling scheme was employed in their selection; they are sites under examination by the authors for many years and with regularly collected spatial video data. The discovery of the GSV spatio-temporal variability occurred only through happenstance as this source of imagery was questioned as a way to fill in data gaps between spatial video field data collection. The second limitation is the validity of the results of this study. As Google replaces GSV data at an unspecified time interval and with no clear geographic pattern, the date disruptions and the dates of data collection are subject to change, therefore the patterns observed in this paper may not hold for the same areas in the future.

## Conclusions

This study indicates that, in the virtual world as in real life, caution should be used when crossing intersections. When using GSV as a replacement for primary field-based data collection, meta-analysis should be conducted, reported, and perhaps even mapped as part of investigations that rely on this source for neighborhood environment surveys. In the five study sites presented in this paper, the majority of disruptions in imagery date occurs within or in close proximity to road intersections and the extent of continuity between these disruptions was often only brief. The short extents of continuity of these data changes means that unless “driving” through one click at a time, the date change can easily be missed and the resulting data that are recorded in the audit may introduce error into all subsequent analyses using these data.

Studies linking place to health face enough difficulties in defining meaningful variables and the pathways through which they operate. For GSV and other secondary sources to contribute to understanding the geographic context of health, it is incumbent upon researchers to use them critically. Meta-analysis as part of standard procedure in using these data can easily be adopted. However, what is also needed is a similar meta-analysis and reporting by the companies that make these data available. In the case of GSV, their methodology is not made public and therefore users are unable to ascertain why the date change disruptions occur as they do. It should be clear to users what streets are covered, when they were covered, when the next data collection will occur, and where to access older versions of data. With these changes, the potential of free publicly available street level imagery to understand spatio-temporal patterns of the built environment on health is promising. Research can then occur with a level of consistency and geographic ubiquity to act as a catalyst for studies on the relationship between places and the health outcomes of the people who live and work in these environments.

## Methods

### Data

Five areas already selected for study of post-disaster recovery were used in this analysis: New Orleans, Louisiana (hurricane); Tuscaloosa, Alabama (tornado); Joplin, Missouri (tornado); Colorado Springs, Colorado (wildfire), and San Diego County, California (wildfire). The study sites represent areas of severe damage that undergo built environment audits at various temporal intervals using the spatial video approach to collect data on spatial patterns of neighborhood recovery [21,24,28]. Though these are relatively small areas (ranging from 2.77 km^2^ to 15.03 km^2^) they are dynamic in showing signs of recovery (rebuilding) and decline (blight) (Table 3). Due to the nature of these changing landscapes, the built environment audits must be conducted relatively frequently and both the date of data collection and the results of coding these data for a specific time period are important building blocks in understanding how places recover after a disaster and the potential relationship between these built environments and health outcomes for returning residents. Given the new GSV date stamp, it was questioned whether a remote survey could be performed to collect current conditions at one point in time. In this process, the frequent disruptions in imagery dates were discovered which led to systematic examination of its incidence across all study sites.

In May 2013 the study sites were virtually “driven” by the authors, progressing one click at a time, using Google Earth Street View. All of the authors are trained in using GSV as a data collection tool in post-disaster environments and have been using this approach since 2008. A total of 281,591.66 m of streets were assessed. Table 3 provides an overview of each of the study areas under investigation. As each street was driven, every time the date of the imagery changed was recorded in the same location on a map. These “disruptions ” were then digitized into ArcGIS 10.1 on TIGER road line files for each of the study sites (Figure 1); these data were used to split and merge the road files based on segments collected on the same date, which led to the formation of a map of “extent of continuity” (Figure 2). These two datasets were created to understand how frequently the date of the imagery changes in each of the study sites, where these changes occur geographically, and how long segments of road lasted for each date stamp (i.e., can we drive entire roads or road segments with a consistent date of imagery?).

### Analysis

#### *Disruption*

From observations recorded while identifying these locations, it was noted that many of the date changes occurred within or near street intersections. Indeed, our first step in investigating the locations of these disruptions in date change was to look for any patterns. In the New Orleans study site we observed a regular pattern that appeared to align with intersections. We then overlayed these disruption locations with streets and a layer of intersection centroids and discovered that this pattern was persistent across study sites. Therefore, a spatial query was employed to measure the distance from each point to its nearest intersection.

#### *Continuity*

In addition to calculating distance from closest intersection for all disruptions, the length of each road segment for each time period was also measured. The TIGER road files were edited so that they were given an attribute named “date” which is the date this section of road has imagery data from GSV. Contiguous line segments with the same date stamp were merged into one line segment. Line segments with different dates of GSV coverage were split by the location of date change.

## Endnotes

^a^In addition to these studies that focus on built environment audits for health, Guo [29], turns to GSV for a different purpose. He employs it with Microsoft Bing StreetSide View and Bing Birds-Eye View to assess on- and off-street parking in the New York City area.

^b^Though Google planned to update imagery on a regular basis, and with higher resolution imagery, what they see as an improvement in data quality poses a major concern to researchers as there is no known archival mechanism for the previous data. Therefore, it may be impossible to return to the imagery used for the audit when it is overwritten with newer, higher resolution imagery. In essence, the raw data upon which these studies are conducted will be lost.

^c^They also propose the following: “Similar to going back to a stored blood spot for biological markers on a respondent, it is possible to return to the Street View images at a later date (provided they have not been updated) if it becomes apparent that other aspects of the environment need to be documented” (5–6).

^d^Information regarding the disruptions of Street View are not made available by Google. The company only provides details about the equipment used, areas covered, and areas they are currently imaging [30]. Google takes the video, date-stamps it, then disassembles the video into individual images, which is what we see on Street View. While the disruptions in date are most likely due to replacement of imagery, Google does not publicize the frequency of this replacement, as this likely varies by locality.

## Competing interests

The authors declare that they have no competing interests.

## Authors’ contributions

JWC conceptualized and designed the study. JWC, AS, and ACi performed data collection. JWC wrote the manuscript and performed analysis. JM contributed to writing the manuscript. ACu and JM provided critical feedback on all versions of the manuscript. All authors read and approved the final manuscript.
